# Preventable Cases of Oral Anticoagulant-Induced Bleeding: Data From the Spontaneous Reporting System

**DOI:** 10.3389/fphar.2019.00425

**Published:** 2019-04-30

**Authors:** Annamaria Mascolo, Rosanna Ruggiero, Maurizio Sessa, Cristina Scavone, Liberata Sportiello, Concetta Rafaniello, Francesco Rossi, Annalisa Capuano

**Affiliations:** ^1^Section of Pharmacology “L. Donatelli”, Department of Experimental Medicine, Campania Pharmacovigilance and Pharmacoepidemiology Regional Centre, University of Campania “Luigi Vanvitelli”, Naples, Italy; ^2^Department of Drug Design and Pharmacology, University of Copenhagen, Copenhagen, Denmark

**Keywords:** oral anticoagulant, bleeding, preventability assessment, spontaneous reporting system, adverse effect

## Abstract

**Background:**

Despite the risk of bleeding is a well-known adverse effect of oral anticoagulants, there is scarce evidence on the preventability of oral anticoagulant-induced bleedings. Therefore, we investigated the potential risk factors related to preventable cases of oral anticoagulant-induced bleedings.

**Methods:**

We performed a study using Individual Case Safety Reports (ICSRs) with an oral anticoagulant as suspected drug among those reported through the spontaneous reporting system of Campania Region from 1 July 2012 to 31 December 2017. The P-method was used for the preventability assessment of all cases of bleeding.

**Results:**

In total, 58 cases out of 253 (22.9%) were preventable, and the most reported suspected drug was an indirect oral anticoagulant (warfarin). Sixty-eight critical criteria for preventability were identified, all related to healthcare professionals’ practices. The most detected risk factor related to healthcare professionals’ practices was the labeled drug–drug interaction for both direct and indirect oral anticoagulants.

**Conclusion:**

Our findings describe the most reported risk factors for preventability of oral anticoagulant-induced bleedings. These factors may be useful for targeting interventions to improve pharmacovigilance activities in our regional territory and to reduce the burden of medication errors and inappropriate prescription.

## Introduction

Oral anticoagulant therapy is widely used for the prevention of stroke and systemic embolism in patients with atrial fibrillation, or for the prevention and treatment of deep vein thrombosis and pulmonary embolism (Raj et al., 1994; Monaco et al., 2017). Oral anticoagulants can be divided into “indirect oral anticoagulants,” like vitamin K antagonists (VKAs; warfarin, or acenocoumarol), which have been the cornerstone of the anticoagulation therapy for decades, and into the more recently approved “direct oral anticoagulants” (DOACs), which are selective inhibitors of single coagulation factors. Belongs to DOACs the reversible direct thrombin inhibitor (dabigatran), and the direct factor Xa inhibitors (rivaroxaban, apixaban, and edoxaban) (Sabir et al., 2014; Monaco et al., 2017). Since their introduction, these more recent drugs have added some advantages to the clinical practice, like the rapid onset of action, the more predictable pharmacokinetics and pharmacodynamics, the potentially reduced risk for drug-drug interactions, and the absence of routine coagulation monitoring (Mekaj et al., 2015; Eikelboom and Merli, 2016; Hicks et al., 2016; Monaco et al., 2017). Thanks to these properties, DOACs represent an effective alternative to VKAs, offering also important safety advantages in terms of risk of bleeding (Eikelboom and Merli, 2016). Accordingly, a large phase III study has shown a consistent reduction of the risk of cerebral hemorrhagic events with DOACs compared to warfarin (Ruff et al., 2014), but DOACs may be associated with a higher risk of gastrointestinal bleedings (Holster et al., 2013; Ruff et al., 2014).

Despite the risk of bleeding is a well-known adverse effect of oral anticoagulants that requires constant monitoring in a real-life context, the evidence on the preventability of oral anticoagulant-induced bleedings is meager. This can be even more interesting considering that among predisposing causes of anticoagulant-induced adverse drug reactions (ADRs), there are medication errors (Piazza et al., 2011; Sessa et al., 2018a) that represent in general an important cause of preventable ADRs (European Medicine Agency, 2013). Moreover, the importance of evaluating the preventability of ADRs is nowadays suggested by both European Medicine Agency and World Health Organization as part of pharmacovigilance-based activities to achieve an effective risk minimization (European Medicine Agency, 2013; Pal et al., 2015). In light of this consideration and taking into account that no study has been conducted in our country to directly evaluate the preventability of bleeding cases reporting an oral anticoagulant as a suspected drug, we decided to conduct a pharmacovigilance study on spontaneous reporting data to fill this gap in knowledge. Therefore, the primary aim of this study was to assess the preventability of bleeding cases reporting an oral anticoagulant as a suspected drug among those sent through the Campania spontaneous reporting system from July 2012 to December 2017.

## Materials and Methods

### Data Source

Individual Case Safety Reports (ICSRs) with an oral anticoagulant as a suspected drug were selected among those reported through the spontaneous reporting system from 1 July 2012 to 31 December 2017. ICSRs were retrieved from the Italian National database for Pharmacovigilance (Rete Nazionale di Farmacovigilanza, RNF). This database collects all ICSRs reported spontaneously or deriving from active pharmacovigilance projects or observational studies. In Italy, every healthcare institution has his own Responsible Person for Pharmacovigilance that provides to send to the RNF each ICSR received from a reporter. We collected for preventability assessment only ICSRs sent to the RNF by one Italian Region (Campania).

### Descriptive and Statistical Analyses

For descriptive purposes, information on age, gender, type of reporter, seriousness, outcome, action taken to solve the ADRs, causality assessment, and number of reported suspected drugs were provided separately for all cases, for all cases of bleeding, and for “preventable” cases of bleeding. Bleeding cases were defined as all cases that reported at least one hemorrhagic adverse event among those described in the ICSR. Cases without hemorrhagic events were classified as not bleeding cases and were analyzed separately. Moreover, ICSRs were classified based on the suspected drug in those reporting an indirect oral anticoagulant (warfarin or acenocoumarol), and those reporting a DOAC (dabigatran, edoxaban, rivaroxaban, or apixaban). All reported preferred terms (PTs) related to bleedings were tabled according to their system organ class (SOC) (Brown et al., 1999). For gastrointestinal bleedings, when their location in the gastrointestinal tract was available in the ICSRs, we classified them into “upper gastrointestinal bleedings” and “lower gastrointestinal bleedings” for each oral anticoagulant. We classified as belonging to the group “upper gastrointestinal bleedings” any hemorrhagic event that occurs between the oropharynx and the ligament of Treitz, or any event that could be highly associated with upper gastrointestinal bleedings (e.g., melena or hematemesis). We classified instead into the group “lower gastrointestinal bleedings” any hemorrhagic event that occurs from the ligament of Treitz to the anus. Reporting odds ratio (ROR), its’ 95% confidence interval (95%CI) and the chi-square test were computed to evaluate if the drugs undervaluation had a lower/higher probability of lower/upper gastrointestinal bleeding when compared to dabigatran. Dabigatran was used as a reference drug considering that clinical evidence has shown a higher risk of lower gastrointestinal bleeding for this drug compared to warfarin (Kolb et al., 2018). For each comparison, a *post hoc* power calculation was performed to assess the achieved statistical power as described by Wang and Chow (2007). For all cases of bleedings, therapeutic indications of oral anticoagulants were tabled. Additionally, the three most reported PTs for each SOC not related to bleeding were tabled. For each ICSR, the seriousness of ADRs was codified as described in the International Council on Harmonization E2D guidelines (European Medicines Agency, 2003), whereas the outcome was categorized into six categories (recovered, improvement, resolution with sequelae, unchanged clinical condition, death, and not available) according to national law. The causality assessment evaluation was performed using the Naranjo algorithm according to the Italian Medicine Agency (AIFA) (Naranjo et al., 1981). Finally, we showed our contribution to the national spontaneous reporting system in terms of pooled ICSRs using the online public report of ADRs (RAM system).

### Preventability Assessment

The P-method was used for the preventability assessment of all cases of bleeding. This method is an algorithm developed by World Health Organization to assess the preventability of ADRs reported in the spontaneous reporting system, and it was validated by pharmacovigilance centers during the WHO Program for International Drug Monitoring (Benkirane et al., 2015). Moreover, it has been validated in several projects of Campania Region that aimed to explore the use of P-method for the preventability assessment of adverse reactions induced by several drug classes (e.g., non-steroidal anti-inflammatory drugs, psychotropic drugs, contrast media, and statins) (Sessa et al., 2016b,c, 2017, 2018b). The P-method based on three steps of evaluation. The first step involved the causality assessment between the drug and the ADR, and we used the Naranjo algorithm. The second step based on the evaluation of the mechanism underlying the ADR in terms of dependency on dose, time, patient susceptibility, or an unknown mechanism. Finally, the last step involved the identification of critical criteria for preventability. The preventability criteria are classified into three sections: healthcare professionals’ practice, product/drug, and patient. There are 16 critical criteria related to the healthcare professionals’ practice, two critical criteria related to the product/drug, and two critical criteria related to the patient. The criteria related to healthcare professionals are incorrect drug dose, administration route, duration, storage, laboratory or clinical monitoring, expired drug, wrong indication, inappropriate prescription for patient’s underlying medical condition or according to the characteristics of the patient, documented hypersensitivity, labeled drug–drug interaction, therapeutic duplication, withdrawal syndrome, and necessary medication not given. The criteria related to the product/drug are poor drug quality and counterfeit drug, and the criteria related to the patient behavior are the non-compliance and self-medication (Benkirane et al., 2015; Sessa et al., 2016c). According to this methodology, a case can be classified as preventable if it is found at least one critical criterion for preventability. In this study, three clinical pharmacologists (one medical doctor and two pharmacists) with multiyear experience in pharmacovigilance and preventability assessment evaluated the preventability of oral anticoagulant-induced bleedings through a case-by-case approach. For the entire evaluation, when the consultation of the Summary of Product Characteristics (SmPC) was needed, those published by the AIFA were used. Details regarding the operative framework for preventability of ICSRs are provided elsewhere (Sessa et al., 2016b,c, 2017).

For all preventable cases of bleeding, the time to event was computed as the difference in days between the onset date of the adverse event and the start date of anticoagulant therapy. A boxplot of time to event was generated for direct and indirect oral anticoagulants. Finally, a cases series of preventable ICSRs was provided.

## Results

In the period from July 2012 to December 2017, 25,609 ICSRs were sent to Campania Pharmacovigilance Regional Centre, of which 453 reported an oral anticoagulant as suspected drug contributing to the 2.4% of national ICSRs (*N* = 18606). The mean age of patients was 72.5 years [standard deviation (SD): 11.2 years], with 52.3% of cases occurred in female patients. In most ICSRs (92.1%), one medical product was reported as a suspected drug. ICSRs were mainly classified by the reporter as not serious (264; 58.3%). Cases classified as serious were for 17.2% related to hospitalizations, for 11.9% registered as clinically significant conditions, for 2.4% defined as life-threatening, and for 1.5% related to the death of the patient. In 66.9% of cases, several actions were taken to solve the ADRs. The main reporter was the physician with 261 (57.6%) out of 453 ICSRs. Causality assessment was probable for 128 (28.3%) cases and possible for 325 (71.7%) cases. Characteristics of cases were presented in Table 1. Because in each ICSR more than one ADR could be reported, we observed a total of 676 ADRs for ICSRs (1.5 suspected ADRs per ICSR). A total of 399 out of 676 ADRs were not related to bleedings. Among them, the six most reported ADRs were abnormal coagulation profile (35/399; 8.8%), epigastric abdominal pain (28/399; 7.0%), dyspepsia (28/399; 7.0%), increased international normalized ratio (INR) (28/399; 7.0%), anemia (21/399; 5.3%), and fluctuating INR (16/399; 4.0%) (Table 2).

**Table 1:** Demographic characteristics and distribution for the type of reporter, drug, documentation, seriousness, outcome, causality assessment, and action taken of cases involving oral anticoagulants recognized in Campania spontaneous reporting system from July 2012 – December 2017.

Variable	Level	Cases of bleeding disorders (*N* = 253)	Preventable cases of bleeding disorders (*N* = 58)	Total (*N* = 453)
Gender	Female	134 (53.0)	36 (62.1)	237 (52.3)
	Male	117 (46.2)	22 (37.9)	212 (46.8)
	Not available	2 (0.8)	–	4 (0.9)
Age	Mean (standard deviation)	72.9 (10.9)	74.4 (10.9)	72.5 (11.2)
Seriousness	Serious–other clinically significant condition	29 (11.5)	5 (8.6)	54 (11.9)
	Serious–death	6 (2.4)	3 (5.2)	7 (1.5)
	Serious–significant or permanent disability	–	–	1 (0.2)
	Serious–hospitalization	55 (21.7)	17 (29.3)	78 (17.2)
	Serious–life threatening	9 (3.6)	3 (5.2)	11 (2.4)
	Not defined	9 (3.6)	–	38 (8.4)
	Not serious	145 (57.3)	30 (51.7)	264 (58.3)
Outcome	Death	6 (2.4)	3 (5.2)	7 (1.5)
	Improvement	93 (36.8)	20 (34.5)	143 (31.6)
	Unchanged clinical condition	18 (7.1)	9 (15.5)	23 (5.1)
	Not available	52 (20.6)	6 (10.3)	129 (28.5)
	Recovered	71 (28.1)	20 (34.5)	134 (29.6)
	Resolution with sequelae	13 (5.1)	–	17 (3.8)
Reporter	Physicians	142 (56.1)	16 (27.6)	261 (57.6)
	Pharmacist	95 (37.5)	40 (69.0)	124 (27.4)
	Other health care professional	8 (3.2)	1 (1.7)	27 (6.0)
	Patient/Citizen or other non-healthcare professional figure	8 (3.2)	1 (1.7)	41 (9.1)
Causality	Possible	152 (60.1)	43 (74.1)	325 (71.7)
	Probable	101 (39.9)	15 (25.9)	128 (28.3)
Action taken	Yes	176 (69.6)	44 (75.9)	303 (66.9)
	No	77 (30.4)	14 (24.1)	150 (33.1)
Oral anticoagulant	Indirect oral anticoagulant	179 (70.8)	53 (91.4)	248 (54.7)
	Warfarin	161 (63.6)	44 (83.0)	223 (49.2)
	Acenocoumarol	18 (7.1)	9 (17.0)	25 (5.5)
	Direct oral anticoagulant	74 (29.2)	5 (8.6)	205 (45.3)
	Dabigatran	35 (13.8)	3 (5.2)	125 (27.6)
	Rivaroxaban	21 (8.3)	1 (1.7)	42 (9.3)
	Apixaban	16 (6.3)	–	35 (7.7)
	Edoxaban	2 (0.8)	1 (1.7)	3 (0.7)
Number of reported suspected drugs	>1	24(9.5)	17 (29.3)	36 (7.9)
	1	229 (90.5)	41 (70.7)	417 (92.1)


**Table 2:** Adverse drug reactions (ADRs) not related to bleeding categorized by system organ class (SOC) and three most reported preferred terms in Individual Case Safety Reports (ICSRs) reporting anticoagulants as suspected drugs.

SOC and ADRs	Number of cases (%)
Gastrointestinal disorders	115 (28.8)
*Epigastric abdominal pain*	28 (7.0)
*Dyspepsia*	28 (7.0)
*Diarrhea*	9 (2.3)
Investigations	95 (23.8)
*Abnormal coagulation profile*	35 (8.8)
*Increased INR*	28 (7.0)
*Fluctuating INR*	16 (4.0)
Nervous system disorders	33 (8.3)
*Headache*	7 (1.7)
*Cerebrovascular accident*	3 (0.8)
*Stroke*	3 (0.8)
Blood and lymphatic system disorders	31 (7.8%)
*Anemia*	21 (5.3)
*Thrombocytopenia*	3 (0.8)
*Pancytopenia*	1 (0.3)
General disorders and administration site conditions	26 (6.5)
*Asthenia*	7 (1.7)
*Therapeutic failure*	4 (1.0)
*Facial edema*	2 (0.5)
Skin and subcutaneous tissue disorders	24 (6.0)
*Itch*	5 (1.2)
*Sweating*	3 (0.8)
*Erythema*	2 (0.5)
Renal and urinary disorders	13 (3.3)
*Renal failure*	6 (1.5)
*Renal lesion*	2 (0.5)
*Decreased renal function*	1 (0.3)
Respiratory, thoracic, and mediastinal disorders	12 (3.0)
*Dyspnea*	8 (2.0)
*Cough*	1 (0.3)
*Respiratory distress*	1 (0.3)
Vascular disorders	12 (3.0)
*Thrombosis*	2 (0.5)
*Hypertension*	2 (0.5)
*Vasculitis*	1 (0.3)
Cardiac disorders	9 (2.3)
*Atrial fibrillation*	4 (1.0)
*Palpitation*	1 (0.3)
*Pericardial effusion*	1 (0.3)
Ear and labyrinth disorders	6 (1.5)
*Vertigo*	4 (1.0)
*Peripheral vertigo*	2 (0.5)
Psychiatric disorders	6 (1.5)
*Drowsiness*	2 (0.5)
*Hallucination*	1 (0.3)
*Agitation*	1 (0.3)
Hepatobiliary disorders	5 (1.2)
*Hypertransaminasemia*	1 (0.3)
*Cholangitis*	1 (0.3)
*Jaundice*	1 (0.3)
Injury, poisoning, and procedural complications	3 (0.8)
*Overdose*	2 (0.5)
*Contusion*	1 (0.3)
Musculoskeletal and connective tissue disorders	3 (0.8)
*Joint effusion*	1 (0.3)
*Hydrarthrosis*	1 (0.3)
*Rhabdomyolysis*	1 (0.3)
Infections and infestations	3 (0.8)
*Rhinopharyngitis*	1 (0.3)
*Infections*	1 (0.3)
*Urinary infection*	1 (0.3)
Eye disorders	1 (0.3)
*Diplopia*	1 (0.3)
Immune system disorders	1 (0.3)
*Allergy*	1 (0.3)
Metabolism and nutrition disorders	1 (0.3)
*Inappetence*	1 (0.3)
Total	399 (100.0)


*INR: international normalized ratio.*

### Cases of Bleeding

A total of 253 cases (55.8%) out of 453 ICSRs reported at least one adverse event related to bleeding. Characteristics of cases associated with bleeding events are presented in Table 1. Specifically, a total of 161 cases related to warfarin, 35 cases to dabigatran, 21 cases to rivaroxaban, 18 cases to acenocoumarol, 16 cases to apixaban, and two cases to edoxaban. The most reported therapeutic indications for direct or indirect oral anticoagulants were atrial fibrillation and antithrombotic prophylaxis (Supplementary Table S1). A total of 277 suspected ADRs were related to bleedings, of which 193 were related to indirect oral anticoagulants. For indirect oral anticoagulant, most adverse events belong to the SOC “respiratory, thoracic, and mediastinal disorders” (87/193; 45.1%), followed by “gastrointestinal disorders” (56/193; 29.0%), “renal and urinary disorders” (20/193; 10.4%), “vascular disorders” (11/193; 5.7%), “nervous system disorders” (11/193; 5.7%), “skin and subcutaneous tissue disorders” (4/193; 2.1%), “injury, poisoning and procedural complications” (2/193; 1.0%), “musculoskeletal and connective tissue disorders” (1/193; 0.5%), and “eye disorders” (1/193; 0.5%). For DOAC, most adverse events belong to the SOC “gastrointestinal disorders” (37/84; 44.0%), followed by “respiratory, thoracic, and mediastinal disorders” (14/84; 16.7%), “renal and urinary disorders” (8/84; 9.5%), “vascular disorders” (8/84; 9.5%), “nervous system disorders” (5/84; 6.0%), “injury, poisoning, and procedural complications” (4/84; 4.8%), “skin and subcutaneous tissue disorders” (3/84; 3.6%), “reproductive system and breast disorders” (3/84; 3.6%), “musculoskeletal and connective tissue disorders” (1/84; 1.2%), and “eye disorders” (1/84; 1.2%) (Table 3). In the evaluation of upper and lower gastrointestinal bleedings for different oral anticoagulants, we found that only 64 of 93 (68.8%) gastrointestinal bleedings met the criteria for classification, of this 35 were related to warfarin, five to acenocoumarol, 14 to dabigatran, five to rivaroxaban, and five to apixaban (Table 4). These bleeding events were mostly distributed into the group “upper gastrointestinal bleeding” for warfarin, acenocoumarol, and apixaban, and into the group “lower gastrointestinal bleeding” for dabigatran and rivaroxaban (Table 4). All cases had both narrative and documental information required for the case evaluation.

**Table 3:** Adverse drug reactions related to bleeding categorized by system organ class (SOC) in Individual Case Safety Reports (ICSRs) reporting oral anticoagulants as suspected drugs.

SOC Reported adverse events	Cases with indirect oral anticoagulants *N* (%)	Cases with direct oral anticoagulants *N* (%)
*Gastrointestinal disorders*	56(29.0)	37(44.0)
Rectal bleeding	15(7.8)	11(13.1)
Bleeding gums	9 (4.7)	3 (3.6)
Melena	14(7.3)	7 (8.3)
Hematemesis	7 (3.6)	1 (1.2)
Bleeding in the digestive tract	4 (2.1)	1 (1.2)
Erosive gastritis	2 (1.0)	–
Abdominal hematoma	1 (0.5)	–
Bleeding in the tongue	1 (0.5)	–
Gastric bleeding	1 (0.5)	2 (2.4)
Gastric ulcer	1 (0.5)	–
Gastrointestinal bleeding	–	3 (3.6)
Intestinal bleeding	–	4 (4.8)
Duodenal ulcer bleeding	1 (0.5)	–
Bleeding of hemorroids	–	1 (1.2)
Hemorrhagic intestinal diverticulitis	–	1 (1.2)
Bleeding in the mouth	–	1 (1.2)
Bleeding from polyps	–	1 (1.2)
*Esophageal ulcer*	–	1 (1.2)

*Respiratory, thoracic, and mediastinal disorders*	87(45.1)	14(16.7)
Epistaxis	78(40.4)	10(11.9)
Hemoptysis	6 (3.1)	2 (2.4)
Blood-tinged sputum	2 (1.0)	–
Coughing up blood	1 (0.5)	1 (1.2)
Hemothorax	–	1 (1.2)

*Renal and urinary disorders*	20(10.4)	8 (9.5)
Hematuria	19(9.8)	7 (8.3)
Hemorrhagic cystitis	1 (0.5)	–
Chromaturia	–	1 (1.2)

*Vascular disorders*	11(5.7)	8 (9.5)
Hematoma	4 (2.1)	2 (2.4)
Hemorrhage	7 (3.6)	5 (6.0)
Hemorrhagic shock	–	1 (1.2)

*Skin and subcutaneous tissue disorders*	4 (2.1)	3 (3.6)
Ecchymosis	3 (1.6)	–
Bleeding of skin ulcer	1 (0.5)	–
Petechia	–	2 (2.4)
Lower extremity ulcer	–	1 (1.2)

*Nervous system disorders*	11(5.7)	5 (6.0)
Cerebral hemorrhage	9 (4.7)	4 (4.8)
Cerebrovascular accident	1 (0.5)	–
Hemorrhagic stroke	1 (0.5)	–
Intracerebral hemorrhage	–	1 (1.2)

*Musculoskeletal and connective tissue disorders*	1 (0.5)	1 (1.2)
Muscle hematoma	1 (0.5)	–
Hemarthrosis	–	1 (1.2)

*Eye disorders*	1 (0.5)	1 (1.2)
Conjunctival hemorrhage	1 (0.5)	1 (1.2)

*Injury, poisoning, and procedural complications*	2 (1.0)	4 (4.8)
Subdural hemorrhage	1 (0.5)	–
Subdural hematoma	1 (0.5)	3 (3.6)
Epidural hematoma	–	1 (1.2)

*Reproductive system and breast disorders*	–	3 (3.6)
Menorrhagia	–	1 (1.2)
Vaginal bleeding	–	1 (1.2)
Intermenstrual bleeding	–	1 (1.2)


**Table 4:** Distribution of upper and lower gastrointestinal bleedings for each oral anticoagulant.

Oral anticoagulant	Upper gastrointestinal bleeding *N* (%)	Lower gastrointestinal bleeding *N* (%)
Warfarin	22 (62.9)	13 (37.1)
Acenocoumarol	4 (80)	1 (20)
Dabigatran	5 (35.7)	9 (64.3)
Rivaroxaban	2 (40)	3 (60)
Apixaban	4 (80)	1 (20)


### Preventability of Cases of Bleeding

**FIGURE 1:**
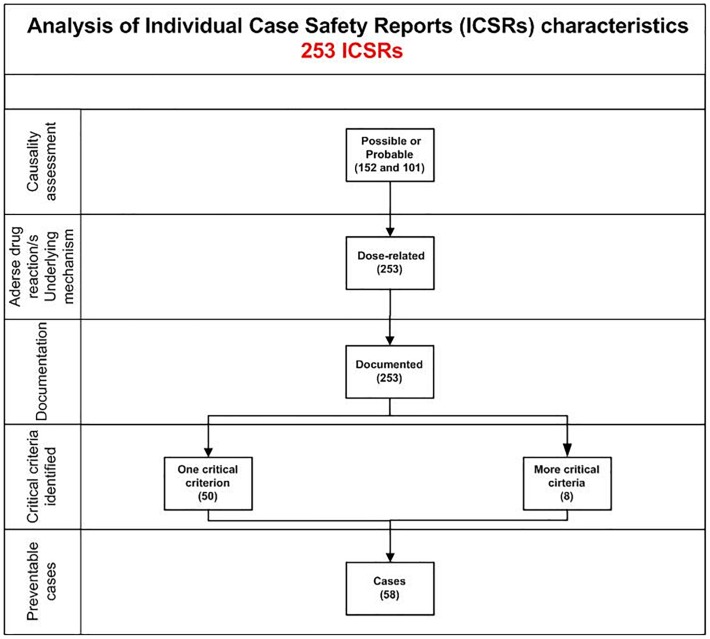
Characteristics of bleeding cases involving oral anticoagulants recognized in Campania spontaneous reporting system from July 2012 to December 2017.

**FIGURE 2:**
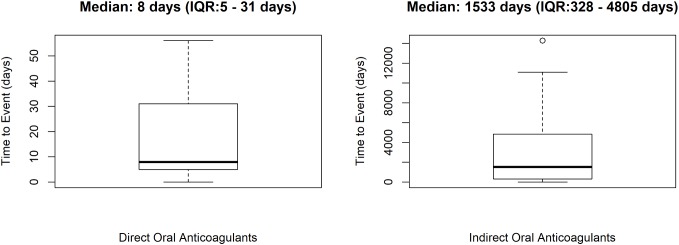
Time to event of preventable cases. ICSRs were screened among those reported in Campania spontaneous reporting system from July 2012 to December 2017.

In total, 58 cases out of 253 (22.9%) were preventable. Preventable cases were mostly reported by healthcare structures located in the province of Avellino (*N* = 39; 67.2%), followed by Napoli (*N* = 8; 13.8%), Caserta (*N* = 7; 12.1%), and Benevento (*N* = 4; 6.9%). A full agreement was reached for all cases by clinical pharmacologists involved in preventability assessment. Considering the hemorrhagic nature of these events, the underlying mechanism of ADRs was classified as dose-related in all cases (Figure 1). Sixty-eight critical criteria related to healthcare professionals’ practices were detected in 58 preventable cases (Supplementary Table S2). Specifically, we observed five different categorizations of the criteria related to health professionals’ practice. In 75.9% of preventable cases, pharmacological and/or non-pharmacological treatments (as action taken) were required to treat the ADRs, of which 23 cases also required drug withdrawal. Characteristics of preventable cases are reported in Table 1 and the clinical description of each case is reported in Supplementary Table S2. The median times to event were 8 days [interquartile range (IQR): 5–31], and 1533 days (IQR: 328–4805) for DOAC and indirect oral anticoagulants, respectively (Figure 2).

#### Preventable Cases Related to the Use of Indirect Oral Anticoagulants

In 53 out of 58 (91.4%) preventable cases, the reported suspected drug was an indirect oral anticoagulant. Specifically, 44 (83.0%) cases reported as suspected drug warfarin and 9 (17.0%) cases reported as suspected drug acenocoumarol. A total of 59 critical criteria related to healthcare professionals’ practices were identified. The most detected critical criteria were labeled drug–drug interactions (50/59; 84.7%), followed by the inappropriate prescription for patient’s underlying medical condition (6/59; 10.2%), and the wrong indication (3/59; 5.1%). A total of 94 drugs were involved in labeled drug–drug interactions. The most reported drug–drug interactions were with proton pump inhibitors (26/50; 52.0%), or statins (15/50; 30.0%). Accordingly, the active ingredients most involved in drug-drug interactions were pantoprazole (9/94; 9.6%), lansoprazole (9/94; 9.6%), allopurinol (7/94; 7.4%), omeprazole (6/94; 6.4%), acetylsalicylic acid (5/94; 5.3%), atorvastatin (5/94; 5.3%), amiodarone (5/94; 5.3%), and simvastatin (4/94; 4.2%) (Supplementary Table S2). The most reported inappropriate prescription for patient’s underlying medical condition was the prescription of an indirect oral anticoagulant in a patient with ulcer (4/6; 66.6%), erosive gastroduodenitis (1/6; 16.7%), or history of cerebral hemorrhage (1/6; 16.7%). The three preventable cases related to a wrong indication were the use of an indirect oral anticoagulant for peripheral obliterating arteriopathy, acute heart failure, or hypertension. Details for each preventable case are provided in Supplementary Table S2.

#### Preventable Cases Related to the Use of Direct Oral Anticoagulants

In five out of 58 (8.6%) preventable cases, the reported suspected drug was a DOAC. Specifically, three (60.0%) cases reported as suspected drug dabigatran, one case rivaroxaban, and one case edoxaban. A total of nine critical criteria were identified. All criteria were related to healthcare professionals’ practices. The most detected critical criteria were labeled drug–drug interactions (3/9; 33.3%), followed by the incorrect dose (3/9; 33.3%), and the inappropriate prescription according to the characteristics of the patient (3/9; 33.3%). The three cases of labeled drug–drug interaction reported as suspected drugs rivaroxaban, dabigatran, and edoxaban. In one case, the labeled drug–drug interaction was the concomitant prescription of rivaroxaban and methimazole in accordance with the SmPC of methimazole. Another case was related to the concominant administration of dabigatran and amiodarone. The last case is the concomitant intake of edoxaban and acetylsalicylic acid in an elderly patient. The critical criteria incorrect dose and inappropriate prescription according to the characteristics of the patient were reported in the same three cases and were all related to the suspected drug dabigatran. In these cases, the patient was treated with 220 mg/day of dabigatran, but according to the SmPC, the recommended dose in elders (>75 years) is 150 mg/day. Details for each preventable case are provided in Supplementary Table S2.

### Comparison of the Reporting Probability of Lower/Upper Gastrointestinal Bleeding Among Oral Anticoagulants

Dabigatran was associated with an increased reporting probability of lower gastrointestinal bleedings rather than upper gastrointestinal bleedings if compared to warfarin (ROR 0.33, 95%CI 0.09–1.19; reference group: dabigatran), acenocoumarol (ROR 0.14, 95%CI 0.01–1.61; reference group: dabigatran), rivaroxaban (ROR 0.83, 95%CI 0.10–6.78; reference group: dabigatran), and apixaban (ROR 0.14, 95%CI 0.01–1.61; reference group: dabigatran). Abovementioned computations were not statistically significant; however, we had no statistical power to detect statistically significant associations as described in Table 5.

**Table 5:** Reporting odds ratio of lower/upper gastrointestinal bleeding with oral anticoagulant with dabigatran as reference drug.

Oral anticoagulant	Control group	Reporting odds ratio (95% confidence interval)	*P*-value	Power (1 - beta error probability)
Warfarin	Dabigatran	0.33 (0.09–1.19)	0.08	0.4
Acenocoumarol	Dabigatran	0.14 (0.01–1.61)	0.08	0.38
Rivaroxaban	Dabigatran	0.83 (0.10–6.78)	0.86	0.05
Apixaban	Dabigatran	0.14 (0.01–1.61)	0.09	0.38


## Discussion

This is the first study conducted in our national territory to assess the preventability of oral anticoagulant-induced bleedings among ICSRs sent through the Campania region (Italy) spontaneous reporting system from 1 July 2012 to 31 December 2017.

Considering the few information available on the preventability of anticoagulant-induced bleedings, especially in Italy, our results may be of great novelty because we were able to provide “real-world evidence” on the quota of preventable ICSRs of bleeding in our Regional territory. Moreover, we provide detailed information on the most reported risk factors for oral anticoagulant-induced bleedings.

In our ICSRs, the mean age was 72.5 years (SD: 11.2 years) in accordance with data showing that in elders anticoagulants are one of main causes responsible for 60% of ADRs leading to hospitalization and 70% of ADRs occurring in hospital (Routledge et al., 2004). In addition, anticoagulants are more likely than other drugs to cause ADRs that result or prolong hospitalization (Davidsen et al., 1988; Bordet et al., 2001), accordingly most of our cases classified as serious reported the criteria “serious – hospitalization.”

Despite in our Region, according to the last national report on drug usage, DOACs are most frequently used than indirect oral anticoagulants (7.2 DDD/1000/inhabitants/day vs. 2.2 DDD/1000/inhabitants/day) (AIFA, 2017), we observed a total of 248 (54.7%) ICSRs that reported an indirect oral anticoagulant as suspected drug. This may be related to the narrow therapeutic window of VKAs compared with DOACs (Fuster et al., 2006).

Overall, from our findings it emerged that abnormalities in the INR, and gastrointestinal abdominal pain were among non-bleeding cases most frequently associated with oral anticoagulants, suggesting that these are the main concerns today by both patients and doctors during anticoagulant therapy. Accordingly, in the literature, the most frequent anticoagulant-associated ADRs are abnormal coagulation test, bleedings, and thrombocytopenia (Piazza et al., 2011). In particular, the occurrence of an excessive anticoagulant effect, such as an increase of INR or of activated partial thromboplastin time (aPTT), has been estimated approximately of 72% (Piazza et al., 2011).

In the analysis of bleedings cases (*N* = 253), the most reported suspected drug was an indirect oral anticoagulant (70.8%). Moreover, we observed that most bleeding events induced by indirect oral anticoagulant belonged to the SOC “respiratory, thoracic, and mediastinal disorders” (45.1%), while for DOACs most adverse events belonged to the SOC “gastrointestinal disorders” (44%). This is in accordance with the higher risk found in scientific literature for the association DOACs and gastrointestinal bleedings (Ruff et al., 2014; Monaco et al., 2017). In the evaluation of upper and lower gastrointestinal bleedings, we were able to classify just 64 ADRs, of which a high number was reported with warfarin and dabigatran. Hemorrhagic events reported with warfarin were more frequently classified as upper gastrointestinal bleedings, while those reported with dabigatran were mostly classified as lower gastrointestinal bleedings. This is in accordance with data of a *post hoc* analysis of the Randomized Evaluation of Long-Term Anticoagulation Therapy (RE-LY) trial that has shown a rate of upper gastrointestinal bleedings similar between dabigatran and warfarin, whereas for lower gastrointestinal bleeding, the rate was higher with dabigatran than warfarin (Kolb et al., 2018). A hypothesis that could explain this finding considers the pharmacokinetic profile of dabigatran etexilate, which is absorbed principally in the stomach and proximal small bowel as an inactive prodrug and then converted to the active form by serum and hepatic esterases. However, the bioavailability of dabigatran is low (3–7%) with an unabsorbed quota being converted to active dabigatran in the distal bowel and then excreted in the feces. This active quota in the distal bowel may be responsible for the onset of lower gastrointestinal bleeding, with a rate even higher than warfarin, which is not activated in the bowel (Feagins and Weideman, 2018).

Different factors are able to influence the risk of gastrointestinal bleeding in patients treated with oral anticoagulants, including the advancing age, concomitant use of acetylsalicylic acid, history of gastrointestinal bleedings, prior/current smokers, atrial fibrillation, and other comorbidities (e.g., renal failure, anemia, diabetes mellitus) (Di Minno et al., 2015). Accordingly, we found that the most reported therapeutic indication for oral anticoagulants was atrial fibrillation and that among concomitant drugs there was the use of acetylsalicylic acid.

For bleedings cases belonging to the SOC “nervous system disorders,” according to literature, we found a lower number of cases related to a DOAC than a VKA (Eikelboom and Merli, 2016).

By applying the P-method, 58 (22.9%) cases of bleedings were preventable, with a total of 68 critical criteria identified. Considering the characteristics of patients in preventable cases, it should be noted that the advanced age was the most reported risk factor for bleedings. In addition, other frequent reported bleeding risk factors were the concomitant clinical conditions like hypertension, diabetes, hepatic or renal damage, and cerebrovascular diseases. Other reported risk factors, although rarely reported, were the prolonged use of warfarin (more than 30 years), the presence of malignancy (multiple myeloma), and hypochromic anemia (AIFA, 2010; Di Minno et al., 2015). All critical criteria were related to healthcare professionals’ practices, and the most reported risk factor was the inappropriate prescription of a drug able to induce drug–drug interactions with oral anticoagulants. In this regard, the potential harm of the so-called “polypharmacy” has been known for some time (Hudson, 1968) being considered as a major medical problem in some countries, and a challenge for the World Health Organization in organizing action programs on multiple therapy (Hogerzeil, 1995). For oral anticoagulants, the potential harm caused by pharmacodynamics and pharmacokinetics interactions with drugs, foods, herbs, and over-the-counter medications was already described in an Italian critical review (Di Minno et al., 2017). Unfortunately, we were able to focus only on drug–drug interaction based on our data source and the preventability tool used. In our results, the most reported drugs involved in drug–drug interactions with an indirect oral anticoagulant were proton pump inhibitors, statins, allopurinol, acetylsalicylic acid, and amiodarone. Accordingly, the evidence suggests a role of the aforementioned drugs as potential risk factors for the development of clinically relevant drug–drug interaction leading to VKA-induced ADRs (Nutescu et al., 2011; Forbes and Polasek, 2017). In accordance with the scientific literature, drugs more involved in drug–drug interaction with DOACs were amiodarone, methimazole, and acetylsalicylic acid (Rafaniello et al., 2016; Forbes and Polasek, 2017; Gelosa et al., 2018). While amiodarone is involved in pharmacokinetic drug–drug interaction as a moderate inhibitor of CYP3A4 and P-gp (Forbes and Polasek, 2017), methimazole may induce a pharmacodynamics interaction through its antivitamin K property (AIFA, 2005). The double effect of antiplatelet and anticoagulant therapy on the bleeding risk is well known, and sometimes well accepted considering the antithrombotic effect achieved by the combined treatment (Dale et al., 1980). In fact, in our preventable case, the co-treatment with apixaban and acetylsalicylic acid has only caused a minor bleeding (epistaxis) in an elderly patient. Moreover, six preventable cases of bleeding with indirect oral anticoagulants were related to the inappropriate prescription of warfarin in patients with hemorrhagic tendencies like an ulcer, erosive gastroduodenitis, or history of cerebral hemorrhage, which are all recognized as part of risk prediction tools for anticoagulant-associated hemorrhages (Shoeb and Fang, 2013).

Interesting, more than half of preventable cases required a pharmacological and/or non-pharmacological treatment to resolve the ADR, presuming a high potential clinical and economic burden on the health care system (Sultana et al., 2013). In this perspective, it seems very important for Competent Authorities to promote the precocious detection of ADRs, and initiatives to quantify their preventable quota in order to reduce where possible the healthcare costs.

We found a different median time to event for direct and indirect oral anticoagulants that was higher for VKAs. One possible explanation for this finding may be the different monitoring of the therapy in patients treated with indirect oral anticoagulants, which are more strictly monitored in the first months of therapy than thereafter. On the contrary, patients in therapy with DOAC are in a fixed-dose regimen since the early start of treatment without any need of routine coagulation monitoring or therapeutic drug monitoring. This may predispose to some dosing error especially in elders. In fact, three of our preventable cases were related to the administration of a dabigatran dose exceeding the maximum recommended dosage in the SmPC for elderly patients.

This study has been promoted by the Campania Pharmacovigilance Regional Centre as part of initiatives to ensure drug safety during the last years (Sessa et al., 2015, 2016a,d; Auricchio et al., 2017; Mascolo et al., 2017; Scavone et al., 2017), and based on our results, other initiatives will be started to establish a proper diagnostic therapeutic plan for the management of anticoagulant therapy and their ADRs, especially in those provinces with the high amount of preventable cases. Moreover, the promotion of appropriate use of these drugs could implicate a massive benefit in terms of public health for our Regional territory, considering also the high morbidity and mortality associated with cardiovascular diseases. Therefore, the appropriate use of drugs like oral anticoagulants may highly affect and reduce cardiovascular risk, becoming highly beneficial for both patients and the National Health Service.

### Strengths and Limitations

Our study has both strengths and limitations. An important strength of this study was the use of a validated tool to quantify preventable cases of ADRs potentially related to oral anticoagulants. However, an important limitation is the lack of generalizability due to the fact that the description of ICSRs characteristics associated with oral anticoagulant takes into account only a small population of an Italian region. Moreover, this study has all the limitations of the spontaneous reporting system, such as underreporting, differential reporting, irregular information quality, and lack of denominator data such as the user population and drug-exposure patterns. For this reason, the population in our study included only oral anticoagulant-induced ADRs patients rather than oral anticoagulant-exposed patients. Furthermore, another important limitation is the lack of information about possible drug–food or drug–herb interactions that cannot be detected for sure through the spontaneous reporting. In fact, this information is not an essential requirement for the spontaneous reporting of suspected ADRs but could represent an important risk factor for the detection of preventable ADRs whether it is added. Therefore, preventability tools that focus also on this aspect should be investigated.

## Conclusion

This is the first study that described preventable cases of oral anticoagulants-related bleedings, of which all cases were related to healthcare professionals’ practices. The most detected critical criterion identified through the application of a validated tool for preventability assessment was the drug-drug interaction. Moreover, 48% of the preventable cases were classified as serious and 69% was associated with a positive outcome. We believe that an improvement of pharmacovigilance activities in our regional territory is needed to put into clinical practice the knowledge of the risk factors for oral anticoagulant-induced bleedings and to reduce the burden of medication errors and inappropriate prescription. Based on our results, several initiatives will be started in the Campania region to share the acquired experiences in routine clinical practice in order to promote the more appropriate use of oral anticoagulant therapy.

## Author Contributions

AM, RR, MS, CS, LS, CR, FR, and AC drafted the work and revised it for important intellectual content, made substantial contributions to the acquisition, analysis, or interpretation of data for the work, approved the final version of the manuscript to be published, and agreed to be accountable for all aspects of the work in ensuring that questions related to the accuracy or integrity of any part of the work are appropriately investigated and resolved. AM and RR developed the concept, designed the study, and wrote the manuscript.

## Conflict of Interest Statement

The authors declare that the research was conducted in the absence of any commercial or financial relationships that could be construed as a potential conflict of interest.
