# Supplementary material for: Preventable Cases of Oral Anticoagulant-Induced Bleeding: Data From the Spontaneous Reporting System
Source: Front Pharmacol. 2019 Apr 30;10:425. doi: 10.3389/fphar.2019.00425 (PMC6503045; doi:10.3389/fphar.2019.00425)
Supplement: Supplementary file 1 [file Table_1.docx]

Supplementary Material

Article Title

Preventable cases of oral anticoagulant-induced bleeding: data from the spontaneous reporting system

**Annamaria Mascolo^1^* & Rosanna Ruggiero^1^, Maurizio Sessa^1,2^, Cristina Scavone^1^, Liberata Sportiello^1^, Concetta Rafaniello^1^, Francesco Rossi^1^ & Annalisa Capuano^1^**

^1^ Campania Pharmacovigilance and Pharmacoepidemiology Regional Centre, Section of Pharmacology “L. Donatelli”, Department of Experimental Medicine, University of Campania “L. Vanvitelli”, Naples, Italy

^2^ Department of Drug Design and Pharmacology, University of Copenhagen, Copenhagen, Denmark

*** Correspondence:**Annamaria Mascolo
annamaria.mascolo@unicampania.it

**Keywords**: oral anticoagulant, bleeding, preventability assessment, spontaneous reporting system, adverse effect.

Supplementary Table 1. Therapeutic indications of oral anticoagulants reported in the individual case safety reports of bleeding, from July 2012 – December 2017.

| **Therapeutic indication** | **Total**  **N** | **Warfarin**  **N** | **Acenocoumarol**  **N** | **Dabigatran**  **N** | **Rivaroxaban**  **N** | **Apixaban**  **N** | **Edoxaban**  **N** |
| --- | --- | --- | --- | --- | --- | --- | --- |
| Atrial fibrillation | 95 | 40 | 9 | 23 | 15 | 8 | 0 |
| Prophylaxis | 94 | 75 | 3 | 9 | 4 | 2 | 1 |
| Not reported | 16 | 7 | 1 | 3 | 1 | 4 | 0 |
| Cardiac fibrillation | 13 | 12 | 0 | 0 | 0 | 0 | 1 |
| Cardiac valve replacement | 9 | 9 | 0 | 0 | 0 | 0 | 0 |
| Deep vein thrombosis | 5 | 5 | 0 | 0 | 0 | 0 | 0 |
| Ischemic cardiomyopathy | 3 | 2 | 0 | 0 | 0 | 1 | 0 |
| Coronary artery disease | 2 | 2 | 0 | 0 | 0 | 0 | 0 |
| Mitral valve replacement | 2 | 0 | 2 | 0 | 0 | 0 | 0 |
| Heart valve disease | 2 | 0 | 2 | 0 | 0 | 0 | 0 |
| Acute heart failure | 1 | 1 | 0 | 0 | 0 | 0 | 0 |
| Arrhythmia | 1 | 1 | 0 | 0 | 0 | 0 | 0 |
| Obliterating thromboangiitis | 1 | 1 | 0 | 0 | 0 | 0 | 0 |
| Stroke | 1 | 1 | 0 | 0 | 0 | 0 | 0 |
| Coagulopathy | 1 | 1 | 0 | 0 | 0 | 0 | 0 |
| Embolism (not specified) | 1 | 0 | 0 | 0 | 1 | 0 | 0 |
| Pulmonary embolism | 1 | 0 | 0 | 0 | 0 | 1 | 0 |
| Hypertension | 1 | 1 | 0 | 0 | 0 | 0 | 0 |
| Pulmonary thromboembolism | 1 | 1 | 0 | 0 | 0 | 0 | 0 |
| Thrombosis (not specified) | 1 | 0 | 1 | 0 | 0 | 0 | 0 |
| Anticoagulant therapy | 1 | 1 | 0 | 0 | 0 | 0 | 0 |
| Peripheral arterial occlusive disease | 1 | 1 | 0 | 0 | 0 | 0 | 0 |

**Supplementary Table 2**. Case series of preventable cases reporting an oral anticoagulant as suspected drug recognized in Campania spontaneous reporting system from July 2012 – December 2017.

| **Case demographic characteristics – Comorbidity  Seriousness – Outcome – Therapeutic indications  Causality Assessment – Adverse Drug Reaction Mechanism** | **Cause of preventability** | **Case description** | **Adverse drug reaction/s** | |
| --- | --- | --- | --- | --- |
| **Case 1 (232379)** Male 60 years old, European – Atrial fibrillation, ischemic cardiomyopathy Serious – hospitalization – Recovered – Antithrombotic prophylaxis Probable Dose-related | Labelled drug-drug interaction | According to the SmPC, concurrent use of warfarin and simvastatin may increase the INR and the risk of bleeding. | Epistaxis | |
| **Case 2 (232513)** Male 87 years old, European Serious – Life threatening –  Unchanged clinical condition – Atrial fibrillation Possible Dose-related | Labelled drug-drug interaction | According to the SmPC, concurrent use of warfarin and heparin, omeprazole, or tamsulosin may increase the INR and the risk of bleeding. Moreover, concomitant use of heparin and warfarin is only possible in emergencies, such as pulmonary embolism, or in the start treatment to achieve an early anticoagulant effect. | Cerebral haemorrhage, drowsiness, abnormal clotting test | |
| **Case 3 (234472)** Male 81 years old, European – Diabetes mellitus, cardiac pacemaker user, atrial fibrillation, coronary artery cardiomyopathy Serious – hospitalization – Recovered – Antithrombotic prophylaxis  Probable Dose-related | Labelled drug-drug interaction | According to the SmPC, concurrent use of warfarin and lovastatin, or tamsulosin may increase the INR and the risk of bleeding. | Hematemesis | |
| **Case 4 (235073)** Male 81 years old, European – Percutaneous transluminal angioplasty, hypertension Serious – life threatening –  Unchanged clinical condition – Atrial fibrillation  Possible Dose-related | Labelled drug-drug interaction | According to the SmPC, concurrent use of acenocoumarol and rosuvastatin, esomeprazole, or acetyl salicylic acid may increase the INR and the risk of bleeding. | Cerebral haemorrhage, dysarthria, hemiplegia, abnormal prothrombin time, drowsiness |  |
| **Case 5 (235541)** Female 73 years old, European – Chronic hepatitis C, cerebral hemorrhage, endocarditis Serious – Life threatening –  Unchanged clinical condition – Cardiac valve replacement Possible Dose-related | Labelled drug-drug interaction  Inappropriate prescription for patient’s underlying medical condition | According to the SmPC, concurrent use of warfarin and pravastatin may increase the INR and the risk of bleeding. In addition, the patient has a history of cerebral haemorrhage, that represents a contraindication to the use of warfarin. | Cerebral haemorrhage, headache, abnormal coagulation test |  |
| **Case 6 (226515)** Female 84 years old, European Not serious – Improvement – Atrial fibrillation Possible Dose-related | Labelled drug-drug interaction | Metimazole may potentiate the anticoagulant activity of rivaroxaban due to its antivitamin K property. | Petechia |  |
| **Case 7 (235553)** Female 78 years old, European –Insertion of cardiac pacemaker, femoral-popliteal arterial bypass, aortic stenosis, erosive gastroduodenitis Serious – hospitalization – Improvement – Atrial fibrillation Possible Dose-related | Labelled drug-drug interaction  Inappropriate prescription for patient’s underlying medical condition | According to the SmPC, concurrent use of warfarin and allopurinol, or pantoprazole may increase the INR and the risk of bleeding.  Moreover, warfarin is contraindicated in case of haemorrhagic tendencies associated with active ulceration or gastrointestinal bleeding. As reported in the ICSR, the patient had erosive gastroduodenitis. | Asthenia, anaemia, melena, gastrointestinal haemorrhage, abnormal coagulation test |  |
| **Case 8 (241259)** Male 84 years old, European – Insertion of pacemakers, hyper-cholesterolemia, hypertension, ulcer Not serious – Improvement – Atrial fibrillation Possible Dose-related | Labelled drug-drug interaction  Inappropriate prescription for patient’s underlying medical condition | According to the SmPC, concurrent use of warfarin and atorvastatin, or lansoprazole may increase the INR and the risk of bleeding.  Moreover, warfarin is contraindicated in case of haemorrhagic tendencies associated with active ulceration or gastrointestinal bleeding. As reported in the ICSR, the patient was treated with lansoprazole for ulcer. | Epistaxis |  |
| **Case 9 (252348)** Male 86 years old, European Serious – hospitalization –  Recovered – Antithrombotic prophylaxis Possible Dose-related | Labelled drug-drug interaction | According to the SmPC, concurrent use of warfarin and diclofenac may increase the risk of bleeding. Moreover, the dose of diclofenac was exceeding the maximum recommended dosage. | Melena, erosive gastritis |  |
| **Case 10 (268507)** Female 77 years old, European Serious – other clinically significant condition –  Recovered – Antithrombotic prophylaxis Possible Dose-related | Labelled drug-drug interaction | According to the SmPC, concurrent use of warfarin and ketoprofen, or betamethasone may increase the INR and the risk of bleeding. | Hematemesis |  |
| **Case 11 (269793)** Female 83 years old, European – Rheumatoid arthritis, hyperuricemia, hypochromic anemia, hypercholesterolemia Not serious – Recovered – Antithrombotic prophylaxis Possible  Dose-related | Incorrect dose  Inappropriate prescription according to the characteristics of the patient | According to the SmPC, patients older than 75 years must receive a low dose of dabigatran (150 mg/day) but in the ICSR the patient was treated with the maximum recommended dosage (220 mg/day). | Haematuria |  |
| **Case 12 (376657)** Female 88 years old, European – Hypertension  Not serious – Recovered – Atrial fibrillation  Possible Dose-related | Incorrect dose  Inappropriate prescription according to the characteristics of the patient | According to the SmPC, patients older than 75 years must receive a low dose of dabigatran (150 mg/day) but in the ICSR the patient was treated with the maximum recommended dosage (220 mg/day). | Rectal haemorrhage |  |
| **Case 13 (270602)** Female 87 years old, European – Insulin-dependent diabetes mellitus, renal failure Serious – hospitalization – Improvement  Possible Dose-related | Labelled drug-drug interaction | According to the SmPC, concurrent use of warfarin and levofloxacin, allopurinol, prednisolone, ceftriaxone, or pantoprazole may increase the INR and the risk of bleeding. | Melena |  |
| **Case 14 (325329)** Female 74 years old, European Serious – other clinically significant condition – Not defined – Antithrombotic prophylaxis  Probable Dose-related | Labelled drug-drug interaction | According to the SmPC, concurrent use of warfarin and pantoprazole may increase the INR and the risk of bleeding. | Haemoptysis |  |
| **Case 15 (430420)** Male 75 years old, European – Hypertension, hypercholesterolemia Not serious – Recovered – Ischemic cardiomyopathy Possible Dose-related | Labelled drug-drug interaction | According to the SmPC, concurrent use of warfarin and acetyl salicylic acid, omeprazole, or simvastatin may increase the INR and the risk of bleeding. | Haemoptysis |  |
| **Case 16 (219631)** Female 80 years old, European Serious – hospitalization – Improvement – Atrial fibrillation Probable Dose-related | Labelled drug-drug interaction | According to the SmPC, concurrent use of warfarin and lansoprazole, or amiodarone may increase the INR and the risk of bleeding. | Subdural haemorrhage, abnormal coagulation test, aphasia |  |
| **Case 17 (337122)** Female 59 years old, European Not serious – Recovered – Antithrombotic prophylaxis Possible Dose-related | Labelled drug-drug interaction | According to the SmPC, concurrent use of warfarin and prednisone may increase the INR and the risk of bleeding. | Epistaxis |  |
| **Case 18 (356786)** Female 87 years old, European – Heart failure Serious – other clinically significant condition – Improvement - Prophylaxis  Possible Dose-related | Labelled drug-drug interaction | According to the SmPC, concurrent use of warfarin and celecoxib, pantoprazole, or acetaminophen /tramadol may increase the INR and the risk of bleeding. | Hematemesis, melena |  |
| **Case 19 (366566)** Male 81 years old, European - Abdominal aneurysm, biliary calculi, chronic renal failure, heart failure, syndrome of asthma and chronic obstructive pulmonary disease, hypertension Serious – hospitalization – Recovered – Atrial fibrillation Possible Dose-related | Labelled drug-drug interaction | According to the SmPC, concurrent use of acenocoumarol and omeprazole may increase the INR and the risk of bleeding. | Duodenal ulcer, subdural hematoma |  |
| **Case 20 (194377)** Female 63 years old, European Not serious – Recovered – Not reported Possible Dose-related | Labelled drug-drug interaction | According to the SmPC, concurrent use of warfarin and ciprofloxacin, or simvastatin may increase the INR and the risk of bleeding. | Haematuria |  |
| **Case 21 (196002)** Male 83 years old, European Not serious – Improvement – Pulmonary thromboembolism Possible Dose-related | Labelled drug-drug interaction | According to the SmPC, concurrent use of warfarin and heparin, or pantoprazole may increase the INR and the risk of bleeding. | Haematuria |  |
| **Case 22 (196153)** Female 81 years old, European Serious – hospitalization – Not defined – Heart valve disease  Probable Dose-related | Labelled drug-drug interaction | According to the SmPC, concurrent use of acenocoumarol and sulfonylureas may increase the INR and the risk of bleeding. | Rectal haemorrhage |  |
| **Case 23 (197049)** Female 54 years old, European Not serious – Recovered – Mitral valve replacement  Probable Dose-related | Labelled drug-drug interaction | According to the SmPC, concurrent use of acenocoumarol and thyroid hormones like levothyroxine may increase the risk of bleeding. | Epistaxis |  |
| **Case 24 (197900)** Female 77 years old, European - Hypertension, heart disease, diabetes, hepatitis C Not serious – Not defined – Atrial fibrillation Possible Dose-related | Labelled drug-drug interaction | According to the SmPC, concurrent use of warfarin and esomeprazole, or atorvastatin may increase the INR and the risk of bleeding. | Muscular haemorrhage |  |
| **Case 25 (197902)** Male 60 years old, European - Atrial fibrillation, ulcer - Serious – hospitalization – Improvement – Heart valve disease Probable  Dose-related | Labelled drug-drug interaction  Inappropriate prescription for patient’s underlying medical condition | According to the SmPC, concurrent use of acenocoumarol and lansoprazole, or atorvastatin may increase the INR and the risk of bleeding.  Moreover, the patient has ulcer that is a contraindication to the acenocoumarol therapy. | Epistaxis, melena, asthenia, anaemia |  |
| **Case 26 (198971)** Female 83 years old, European Serious – hospitalization – Improvement – Atrial fibrillation Possible Dose-related | Labelled drug-drug interaction | According to the SmPC, concurrent use of warfarin and allopurinol may increase the INR and the risk of bleeding. | Rectal haemorrhage |  |
| **Case 27 (202834)** Female 71 years old, European Serious – other clinically significant condition – Recovered - Peripheral arterial occlusive disease  Possible Dose-related | Wrong indication | The indication peripheral obliterating arteriopathy is not reported in the SmPC of warfarin. | Bleeding gums |  |
| **Case 28 (203189)** Male 48 years old, European - Heart failure, ischemic heart disease, hypertension Serious – death – Death – Ischemic cardiomyopathy Possible Dose-related | Labelled drug-drug interaction | According to the SmPC, concurrent use of warfarin and allopurinol, lansoprazole, or acetyl salicylic acid may increase the INR and the risk of bleeding. | Epistaxis, rhabdomyolysis, renal failure, thrombocytopenia |  |
| **Case 29 (204998)** Male 80 years old, European Not serious – Recovered – Atrial fibrillation Possible Dose-related | Labelled drug-drug interaction | According to the SmPC, concurrent use of warfarin and pentoxifylline may increase the INR and the risk of bleeding. | Epistaxis |  |
| **Case 30 (205305)** Female 90 years old, European Serious – hospitalization – Improvement – Acute heart failure Probable Dose-related | Wrong indication | The indication acute heart failure is not reported in the SmPC of warfarin. | Bleeding |  |
| **Case 31 (206080)** Female 80 years old, European Serious – death – Death – Atrial fibrillation Possible Dose-related | Labelled drug-drug interaction | According to the SmPC, concurrent use of warfarin and diclofenac, ketorolac, or omeprazole may increase the INR and the risk of bleeding. | Cerebral haemorrhage |  |
| **Case 32 (206081)** Female 84 years old, European – Acute pancreatitis, cholelithiasis, colon diverticulosis, intestinal polyps Serious – hospitalization – Improvement – Atrial fibrillation Possible Dose-related | Labelled drug-drug interaction | According to the SmPC, concurrent use of warfarin and lansoprazole may increase the INR and the risk of bleeding. | Melena, rectal haemorrhage |  |
| **Case 33 (206217)** Female 66 years old, European - Ischemic stroke, hypertension Serious – death – Death – Atrial fibrillation  Possible Dose-related | Labelled drug-drug interaction | According to the SmPC, concurrent use of warfarin and ranitidine may increase the INR and the risk of bleeding. | Cerebral haemorrhage |  |
| **Case 34 (207642)** Male 60 years old, European - Transurethral resection of the prostate, aortic aneurysm Not serious – Recovered – Cardiac valve replacement Possible Dose-related | Labelled drug-drug interaction | According to the SmPC, concurrent use of warfarin and pantoprazole, acetyl salicylic acid, or rosuvastatin may increase the INR and the risk of bleeding. | Haematuria |  |
| **Case 35 (207462)** Female 54 years old, European Not serious – Improvement – Mitral valve replacement Possible Dose-related | Labelled drug-drug interaction | According to the SmPC, concurrent use of acenocoumarol and thyroid hormones like levothyroxine may increase the INR and the risk of bleeding. | Epistaxis |  |
| **Case 36 (209482)** Female 59 years old, European - Cardiac valvulopathy, gastric ulcer Serious – hospitalization – Unchanged clinical condition  Possible – Atrial fibrillation Dose-related | Labelled drug-drug interaction  Inappropriate prescription for patient’s underlying medical condition | According to the SmPC, concurrent use of acenocoumarol and nonsteroidal anti-inflammatory drugs like ibuprofen may increase the INR and the risk of bleeding.  Moreover, acenocoumarol is contraindicated in patient with duodenal ulcer. | Melena, increased INR |  |
| **Case 37 (210238)** Male 78 years old, European - Benign prostatic hyperplasia Not serious – Improvement – Atrial fibrillation Probable Dose-related | Labelled drug-drug interaction | According to the SmPC, concurrent use of warfarin and allopurinol, or simvastatin may increase the INR and the risk of bleeding. | Haematuria |  |
| **Case 38 (210239)** Male 66 years old, European - Coronary artery bypass, atrial fibrillation, diabetes Serious – hospitalization – Unchanged clinical condition – Cardiac valve replacement Probable Dose-related | Labelled drug-drug interaction | According to the SmPC, concurrent use of warfarin and omeprazole, ciprofloxacin, or zafirlukast may increase the INR and the risk of bleeding. | Anaemia, gastrointestinal bleeding, melena |  |
| **Case 39 (214830)** Male 64 years old, European Not serious – Unchanged clinical condition – Cardiac valve replacement Possible Dose-related | Labelled drug-drug interaction | According to the SmPC, concurrent use of warfarin and omeprazole, or allopurinol may increase the INR and the risk of bleeding. | Haematuria, increased INR |  |
| **Case 40 (214710)** Male 59 years old, European Not serious – Recovered – Atrial fibrillation Probable Dose-related | Labelled drug-drug interaction | According to the SmPC, concurrent use of warfarin and amiodarone, rosuvastatin, or lansoprazole may increase the INR and the risk of bleeding. | Haematuria, increased INR |  |
| **Case 41 (220844)** Female 66 years old, European –Raynaud's disease, amputation Not serious – Recovered - Thrombosis Possible Dose-related | Labelled drug-drug interaction | According to the SmPC, concurrent use of acenocoumarol and pentoxifylline may increase the INR and the risk of bleeding. | Haemoptysis, abnormal coagulation test |  |
| **Case 42 (216447)** Female 81 years old, European Serious – hospitalization – Improvement – Atrial fibrillation  Possible Dose-related | Labelled drug-drug interaction | According to the SmPC, concurrent use of warfarin and heparin may increase the INR and the risk of bleeding. | Rectal haemorrhage |  |
| **Case 43 (216510)** Female 83 years old, European - Ascites, colon diverticulosis, liver disease Serious – hospitalization – Improvement – Atrial fibrillation  Possible Dose-related | Labelled drug-drug interaction | According to the SmPC, concurrent use of warfarin and diclofenac, allopurinol, atorvastatin, or pantoprazole may increase the INR and the risk of bleeding. | Asthenia, anaemia, dyspnoea, gastrointestinal haemorrhage, increased INR, melena |  |
| **Case 44 (220222)** Female 53 years old, European Not serious – Recovered - Hypertension  Probable Dose-related | Wrong indication | The indication hypertension is not reported in the SmPC of warfarin. | Epistaxis |  |
| **Case 45 (217835)**  Male 74 years old, European - Tubercolosis Not serious – Improvement – Cardiac valve replacement Possible Dose-related | Labelled drug-drug interaction | According to the SmPC, concurrent use of warfarin and isoniazid, lansoprazole, or tamsulosin may increase the INR and the risk of bleeding. | Epistaxis, abnormal coagulation test |  |
| **Case 46 (220405)** Male 81 years old, European - Hypertensive crisis Not serious – Improvement – Atrial fibrillation Possible Dose-related | Labelled drug-drug interaction | According to the SmPC, concurrent use of acenocoumarol and thyroid hormones like levothyroxine may increase the risk of bleeding. | Epistaxis |  |
| **Case 47 (221575)** Female 89 years old, European Not serious – Recovered – Atrial fibrillation  Possible  Dose-related | Labelled drug-drug interaction | According to the SmPC, concurrent use of warfarin and ketoprofen may increase the INR and the risk of bleeding. | Epistaxis |  |
| **Case 48 (221577)** Female 89 years old, European – Stroke, diabetes Not serious – Recovered – Atrial fibrillation  Possible Dose-related | Labelled drug-drug interaction | According to the SmPC, concurrent use of warfarin and ketoprofen may increase the INR and the risk of bleeding. | Epistaxis |  |
| **Case 49 (221760)** Female 75 years old, European – Hypertensive crisis, systemic lupus erythematosus Not serious – Improvement – Deep vein thrombosis  Possible Dose-related | Labelled drug-drug interaction | According to the SmPC, concurrent use of warfarin and pantoprazole, or phenobarbital may increase the INR and the risk of bleeding. | Epistaxis |  |
| **Case 50 (222953)** Female 67 years old, European – Dysarthria, hypertension Not serious – Unchanged clinical condition – Atrial fibrillation Probable Dose-related | Labelled drug-drug interaction | According to the SmPC, concurrent use of warfarin and amiodarone, lansoprazole, or atorvastatin may increase the INR and the risk of bleeding. | Epistaxis, abnormal coagulation test |  |
| **Case 51 (223904)** Female 89 years old, European – Hypertension  Not serious – Improvement –Cardiac fibrillation  Possible Dose-related | Labelled drug-drug interaction | According to the SmPC, concurrent use of warfarin and lansoprazole may increase the INR and the risk of bleeding. | Epistaxis, abnormal coagulation test |  |
| **Case 52 (224490)** Male 64 years old, European – Angioplasty  Not serious – Not defined – Cardiac Fibrillation Possible Dose-related | Labelled drug-drug interaction | According to the SmPC, concurrent use of warfarin and lovastatin, or acetyl salicylic acid may increase the INR and the risk of bleeding. | Haematuria |  |
| **Case 53 (226514)** Female 87 years old, European – Duodenal ulcer, diverticulosis, hepatitis B Not serious – Not defined – Atrial fibrillation Possible Dose-related | Labelled drug-drug interaction  Inappropriate prescription for patient’s underlying medical condition | According to the SmPC, current use of warfarin and acetyl salicylic acid may increase the INR and the risk of bleeding.  Moreover, warfarin is contraindicated in patient with duodenal ulcer. | Rectal haemorrhage, abnormal coagulation test |  |
| **Case 54 (226599)** Female 60 years old, European – Dermatomyositis, chronic renal failure, pulmonary embolism, diabetes- Serious – hospitalization – Unchanged clinical condition – Atrial fibrillation Possible Dose-related | Labelled drug-drug interaction | According to the SmPC, concurrent use of warfarin and propafenone, pantoprazole, prednisone, and trimethoprim/sulfamethoxazole may increase the INR and the risk of bleeding. | Epistaxis, abnormal coagulation test, acute renal failure |  |
| **Case 55 (226607)** Female 72 years old, European – Multiple myeloma, Thompson hip prosthesis Serious – other clinically significant condition – Not defined – Cardiac valve replacement Possible Dose-related | Labelled drug-drug interaction | According to the SmPC, concurrent use of warfarin and levothyroxine may increase the INR and the risk of bleeding. | Rectal haemorrhage, abnormal coagulation test |  |
| **Case 56 (426664)** Female 81 years old, European – Atrial fibrillation, hypertension, cerebral stroke, non-Q wave myocardial infarction Not serious – Recovered - Prophylaxis Possible Dose-related | Labelled drug-drug interaction | According to the SmPC, concurrent use of edoxaban and acetyl salicylic acid in elderly patients may increase the risk of bleeding. | Epistaxis, drug-drug interaction |  |
| **Case 57 (230503)** Male 83 years old, European Not serious – Recovered – Antithrombotic prophylaxis Possible Dose-related | Labelled drug-drug interaction  Incorrect dose  Inappropriate prescription according to the characteristics of the patient | According to the SmPC, concurrent use of dabigatran and amiodarone may increase the INR and the risk of bleeding.  According to the SmPC, patients older than 75 years must receive a low dose of dabigatran (150 mg/day) but in the ICSR the patient was treated with the maximum recommended dosage (220 mg/day). | Haematuria |  |
| **Case 58 (231382)** Female 70 years old, European – Haemorrhoid Not serious – Improvement – Atrial fibrillation Possible Dose-related | Labelled drug-drug interaction | According to the SmPC, concurrent use of warfarin and amiodarone may increase the INR and the risk of bleeding. | Rectal haemorrhage, abnormal coagulation test |  |

*INR: International Normalized Ratio; ICSR: Individual Case Safety Report; SmPC: Summary of product characteristics.*
